# An indoor radio mapping dataset combining 3D point clouds and RSSI

**DOI:** 10.1016/j.dib.2026.112959

**Published:** 2026-06-11

**Authors:** Ljupcho Milosheski, Kuon Akiyama, Blaž Bertalanič, Jernej Hribar, Ryoichi Shinkuma

**Affiliations:** a Jožef Stefan Institute, Department of Communication Systems, Ljubljana, 1000, Slovenia; b International Postgraduate School Jožef Stefan, Information and Communication Technologies, Ljubljana, 1000, Slovenia; c Faculty of Engineering, Shibaura Institute of Technology, 3 Chome-7-5 Toyosu, Koto City, Japan

**Keywords:** Indoor radio mapping, LiDAR, RSSI, Wireless, Dataset

## Abstract

The growing number of smart devices supporting bandwidth-intensive and latency-sensitive applications, such as real-time video analytics, smart sensing, Extended Reality (XR), etc., necessitates reliable indoor wireless connectivity. In such environments, accurate Radio Environment Maps (REMs) enable adaptive wireless network planning and optimization of Access Point (AP) placement. However, generating realistic REMs remains difficult due to the variability of indoor environments and the limitations of existing modelling approaches, which often rely on simplified layouts or synthetic data. These challenges are further amplified by the adoption of next-generation Wi-Fi standards, operating at higher frequencies with limited range and wall penetration. To support progress in this area, we collected a dataset that combines high-resolution 3D LiDAR scans with Wi-Fi RSSI measurements across 20 setups in a multi-room indoor environment. It includes two measurement scenarios, one with and one without human presence, enabling development and validation of REM estimation models that incorporate physical geometry and environmental dynamics. The described dataset supports research in data-driven wireless modelling and the development of high-capacity indoor communication networks.

Specifications Table

**Table:** 

Subject	Computer Sciences
Specific subject area	Radio Environment Map estimation based on 3D representation of the operating environment.
Type of data	.ply (point cloud), .csv (raw user equipment measurements), .h5 (combined Received Signal Strength Indicator values and measurement locations)
Data collection	The dataset consists of high-resolution 3D LiDAR point clouds of an indoor environment and corresponding Wi-Fi RSSI measurements collected using a single access point. Measurements were acquired in two distinct indoor areas (office and corridor) under both unoccupied and occupied conditions, capturing the impact of geometry and human presence on signal propagation. The final dataset includes 20 unique AP setups and enables joint analysis of spatial geometry and real-world indoor radio propagation.
Data source location	Faculty of Engineering, Shibaura Institute of Technology, 3 Chome–7–5 Toyosu, Koto City, Japan.
Data accessibility	Repository name: An Indoor Radio Mapping Dataset Combining 3D Point Clouds and RSSI Data identification number: 10.5281/zenodo.20411480 Direct URL to data: https://doi.org/10.5281/zenodo.20411480
Related research article	None

## 1 Value of the Data

• The dataset enables the development and evaluation of self-supervised and generative machine learning models for Radio Environment Map (REM) estimation by jointly leveraging high-resolution 3D point cloud data and real Wi-Fi RSSI measurements in indoor environments with and without human presence.
• To the best of our knowledge, this is the first dataset combining 3D point clouds with RSSI measurements from an indoor environment.
• By combining detailed indoor geometry with spatially aligned signal measurements, the dataset supports research on technology-agnostic radio propagation modelling, facilitating studies that link physical environment structure to electromagnetic signal behaviour.
• Measurements collected in both unoccupied and realistically occupied scenarios allow analysis of the impact of human presence and environmental variability on indoor Wi-Fi coverage, which is critical for applications requiring high reliability, throughput, and low latency (e.g., XR).
• The availability of distinct indoor layouts (office and corridor) and flexible usage configurations enables investigation of diverse propagation conditions, including open spaces, long line-of-sight corridors, reflection-dominated environments, and multi-wall obstruction scenarios.
• The accompanying open-source toolbox for point cloud alignment, visualization, and Wi-Fi data processing lowers the barrier to reuse, and supports reproducible experimentation with REM construction and indoor spectrum analysis workflows.

## 2 Background

Smart devices such as cameras, doorbells and voice assistants like Alexa have become an integral part of our daily lives, as they make everyday tasks much easier and increase productivity. To provide smart services, these devices require a continuous internet connection and their bandwidth requirements are expected to increase with the integration of advanced features such as high-definition video streaming and real-time Artificial Intelligence (AI) inference tasks. To fulfil these requirements in an indoor environment, wireless technologies such as Wi-Fi are particularly well suited due to their high data rates, cost efficiency and adaptability. However, optimising Wi-Fi performance in complex indoor environments remains a challenge due to factors such as signal attenuation from walls, interference from household appliances and dynamic physical obstructions. This becomes even more urgent with the rise of Extended Reality (XR) technologies, e.g., Virtual Reality (VR), Mixed Reality (MR), Augmented Reality (AR) [1], etc., which impose far more stringent connectivity requirements such as ultra-low latency and extremely high throughput [2]. Their demands often exceed those of conventional smart devices and expose the limitations of existing Wi-Fi deployments, particularly when Access Points (APs) are suboptimally placed. The challenge is further amplified with the deployment of newer standards like Wi-Fi 7 (IEEE 802.11be) and the upcoming Wi-Fi 8 (IEEE 802.11bn), whose use of higher frequencies results in reduced wall penetration and limited range.

Consequently, the development of a comprehensive dataset documenting Wi-Fi propagation characteristics in indoor environments, for example homes with multiple rooms or office spaces, is critical. Such datasets will enable the construction of detailed indoor Radio Environment Maps (REMs), support the development of predictive channel models and AI-driven network optimization tools, and inform the strategic placement of APs to sustain high-performance connectivity. This is particularly important for the design of XR-ready environments where immersive and seamless experiences must be reliably maintained in real time.

Various approaches for REMs have been proposed in the literature, which are crucial for understanding spatial signal distribution and optimising wireless deployment. The classical techniques can be broadly categorised into direct methods, which spatially interpolate signal measurements [3], and indirect methods, which are based on known or estimated transmission parameters. Among the most established are ray-tracing-based models implemented in tools such as Wireless InSite [4] and NVIDIA’s Sionna [5], which simulate electromagnetic propagation using geometric and physical scene representations. These methods are particularly useful for generating large synthetic datasets by varying the placement of APs and environmental configurations, providing a valuable basis for training supervised learning models. In parallel, Machine Learning (ML) based approaches have gained significant attention due to their ability to learn complex propagation patterns from data. Classical ML methods such as Support Vector Machines (SVMs) and gradient boosting algorithms such as XGBoost [6] have been used for REM construction, while more recently deep learning techniques such as Generative AI (GAI) [7], Large Language Models (LLMs) [8] and Graph Neural Networks (GNNs) [9], have shown promise for learning high-dimensional mappings between environmental inputs and signal properties. In particular, GAI has shown the potential to combine heterogeneous data modalities, such as geometric information and wireless signal features, which is crucial for realistic REM generation. While many of these methods have been evaluated in both outdoor [10,11] and indoor [12,13] environments, the lack of publicly available, high-resolution datasets remains a significant limitation in developing advanced methods for optimising wireless deployments. Existing datasets often lack the spatial diversity required for modeling complex indoor propagation, especially for high performance applications such as XR.

Combining Received Signal Strength Indicator (RSSI) measurements with detailed 3D point cloud data offers a way to overcome this gap by embedding the physical geometry directly into the signal prediction. This representation enables learning-based models to account for occlusions, materials and structural features that influence signal behaviour, supporting the development of more accurate, robust and generalisable REM estimation systems tailored to future wireless connectivity requirements.

REM models development for indoor use cases, as a target purpose of the data published in this paper, is typically solved based on floor plans and material properties [13], considering a simpler, top-view 2D scenario. Recently, more detailed 3D data of the environments [12], such as furniture and room geometry, are also part of the input, thus providing the advantage of containing fine-grain specifics of the 3D environment. However, the developed indoor models often rely on purely simulated datasets for training and performance validation due to the labor-intensive process of performing actual measurements and the necessity of large datasets for training. While ray-tracing tools could provide highly realistic simulated data in large quantities, thus solving the issue of the data requirement, they cannot fully replicate the complex and dynamic nature of real indoor signal propagation.

In this paper, we describe a dataset that combines high-resolution 3D point clouds of an indoor environment, captured using LiDAR sensors [14], and corresponding Wi-Fi RSSI measurements, using single AP. The 3D scans can be used to construct geometrically accurate models, compatible with simulation frameworks such as Mitsuba renderer [15], supporting hybrid approaches that combine real and synthetic data.

## 3 Data Description

**Fig. 1:**
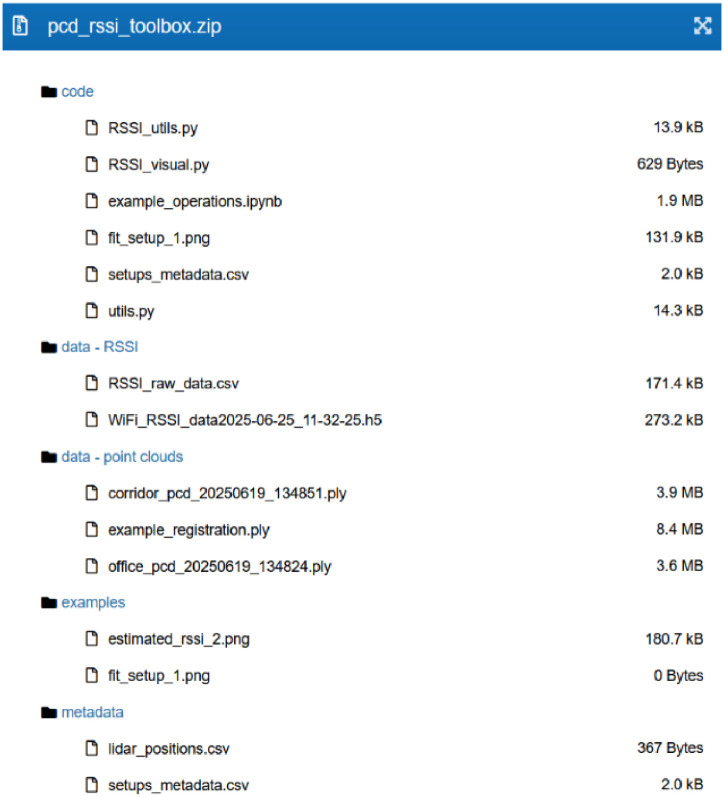
Organization of the dataset repository.

The deposited data^1^ consists of the 3D point clouds and Wi-Fi RSSI measurements, organized as shown in Fig. 1. The 3D point clouds are provided as three ***.ply*** files, which include separate scans of the office and the adjacent corridor that together cover the measurement area, and one example output of the point cloud registration, (spatial alignment of the two scans) produced by the ***example_operations.ipynb*** script. Further details of the acquisition and registration are given in the *3D data* subsection below. The Wi-Fi RSSI measurements are provided in two complementary formats, namely a .csv file with the raw per-measurement records preserving all original Wi-Fi Analyzer fields, and a .h5 file with the same measurements compiled into a structured 3D array indexed by setup and spatial raster cell, with full details given in the *RSSI data* subsection below.

### 3.1 3D data

**Fig. 2:**
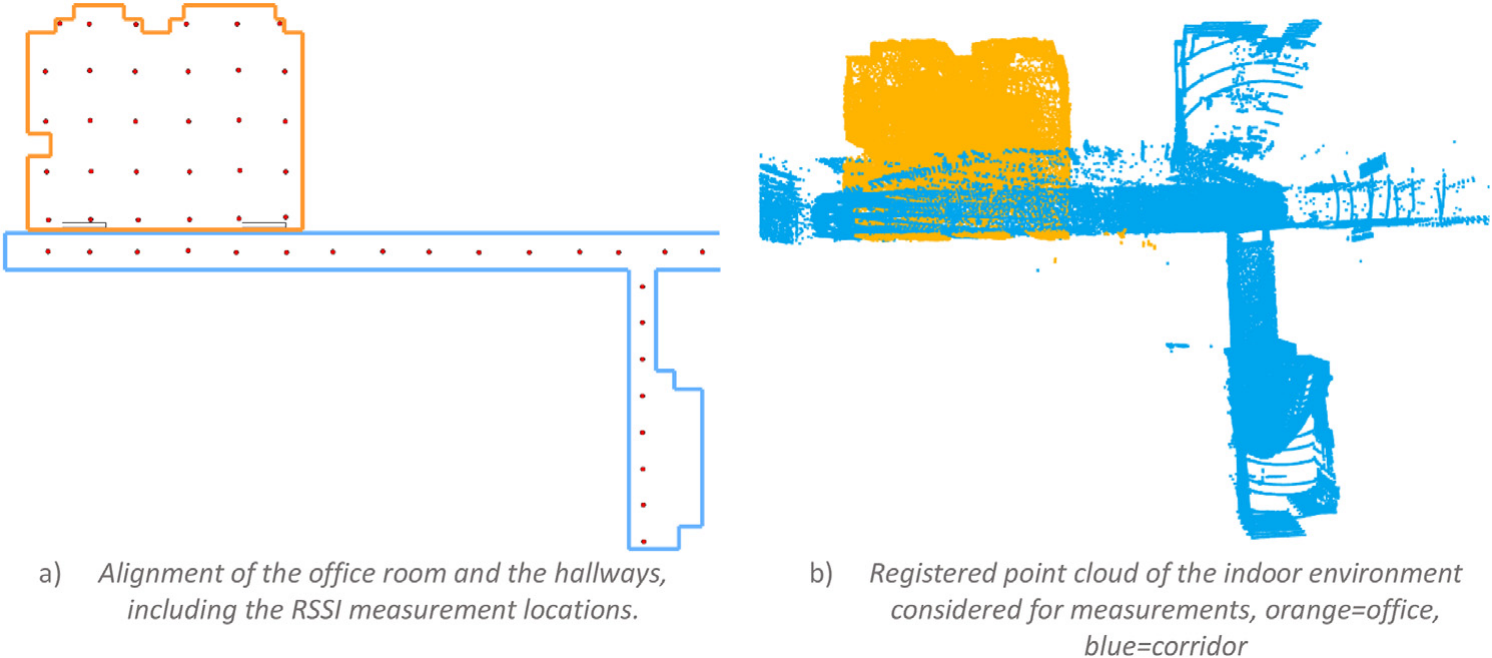
Indoor environment setup and registered point cloud.

The point cloud data is provided in three separate files: one containing the office data, another containing the corridor data, and a third. ***ply*** file representing the combined point cloud. This combined model accurately reflects the physical environment in which the RSSI measurements were collected and is produced through a registration process that aligns the two individual point clouds. Registration is performed manually by selecting corresponding points between the office and corridor datasets, which in this case are sampled from the common wall that separates them. A total of 8 correspondence points were manually selected on the shared glass wall to compute the rigid alignment between the office and corridor point clouds. In Fig. 2a we show the top view of the office layout and an example results of the registration process, combining the two separately acquired point clouds, are shown in Fig. 2b Points corresponding to the office are marked in orange, while those representing the corridor and elevator area are marked in blue. The overlapping regions in the figure result from mutual visibility through the glass wall separating the two spaces. Because the LiDAR measures distances to reflective surfaces, it captured corridor points during the office scan and office points during the corridor scan, as the laser beam passed through the glass and reflected off the walls behind it. As the raw 3D data includes a significant number of outlier points, additional post-processing may be necessary when used for generating precise 3D models of the environment. To support such tasks, we also provide a lightweight toolbox for basic point cloud operations such as loading, visualization, outlier removal, and manual registration. In total, the office scan comprises 148,850 points and the corridor scan 163,945 points, giving 312,795 points in the registered (combined) point cloud (Table 1).

**Table 1:** Structure of the .h5 file documenting the four stored fields, their shapes, dtypes, units, and contents.

Field	Shape	Dtype	Units	Description
data	(20, 28, 30)	float64	dBm	RSSI values per setup over a 28 × 30 spatial raster at 1 m resolution; only the 53 measurement positions (on a 2 m grid) are populated, intermediate cells are 0.
indices	(20, 28, 30)	float64	–	Measurement-point identifier (1–53) at the corresponding raster cells (0 elsewhere); mirrors the spatial structure of data.
ap_locations	(20,)	int64	position index	Measurement-point indices (1–53) used as AP placement locations across the 20 setups.
setup	(20,)	int64	–	Integer setup identifier (1–20) for each of the 20 AP configurations.

### 3.2 RSSI data

**Fig. 3:**
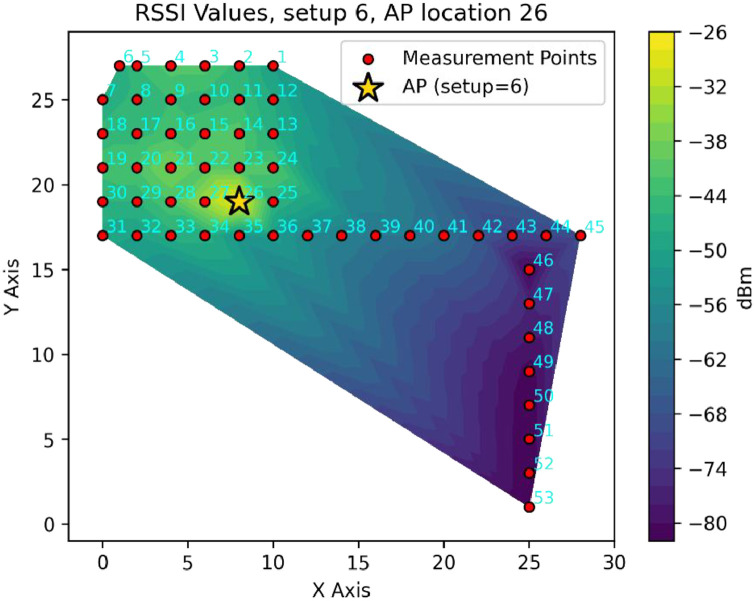
Example plot of single measurement setup.

The RSSI data is available in two separate formats. The first one is a **.csv** file (visualized in Table 2) containing the raw measurements of the RSSI, together with other AP-specifics, such as estimated distance to the AP, exact frequency, channel, etc. In another ***.h5*** file, with contents: the extracted RSSI measurements (*data*) are in matrix format as visualized in Fig. 3, allowing for direct operation on the RSSI data. Furthermore, labels, regarding the setup number (***setup***), AP location (***ap-locations***), and measurement flow (***indices***) are also included in the ***.h5*** file. Regarding the potential reuse, the code for extracting the RSSI data and some simple manipulations, such as visualization of particular setups and filling missing values, will also be provided as part of the toolbox. Missing data appear mostly on the outermost data acquisition points, such as 50–53 in Fig. 3, due to the excessive distance and multiple walls between the AP and UE.

**Table 2:** Visualization of a truncated sample of the .csv file.

#	Time Stamp	SSID	…	Fast Roaming	Setup
0	2024/10/21–11:21:51	TP-Link_4462	…	\n	1
1	2024/10/19–10:14:38	TP-Link_4462	…	\n	1
2	2024/10/19–10:15:45	TP-Link_4462	…	\n	1
3	2024/10/19–10:16:09	TP-Link_4462	…	\n	1
4	2024/10/19–10:16:41	TP-Link_4462	…	\n	1
…

For clarity on the file structures, the RSSI values are reported in dBm, the *data* and *indices* fields in the .h5 file are 3D arrays of shape (20, 28, 30), indexed as [setup k, row i, column j], where the first axis indexes the 20 AP setups and the remaining axes index a 28 × 30 spatial raster at 1 m resolution covering the region of interest. The 53 actual measurement positions (on a 2 m grid) are populated, while the intermediate cells are empty (0) and are filled by interpolation only when the toolbox is used for visualisation or REM validation. *ap_locations* lists all possible AP placement locations across the 20 setups, given as measurement-point indices (1–53), where each AP location coincides with one of the measurement points. The mapping from these indices to metric coordinates is given by the 2 m grid in Fig. 2a (see also the RSSI Measurement Process section). *indices* mirrors the spatial structure of data and stores the measurement-point identifier (1–53) at the corresponding raster cells (0 elsewhere); and *setup* stores the integer identifier (1–20) of each AP configuration. For example, indices[k, i, j] = 47 indicates that the RSSI value stored at data[k, i, j] corresponds to measurement point 47. The accompanying RSSI_raw_data.csv contains one row per individual measurement and preserves all original Wi-Fi Analyzer fields, including the RSSI value (dBm), channel frequency (MHz), channel number, link speed, estimated distance, and the setup identifier. The three point cloud files contain only XYZ coordinates in millimetres (no colour or intensity attributes), and are provided as the raw merged scans without any additional processing applied. The point-cloud coordinates are expressed in millimetres, whereas all distances and dimensions reported in the main text and in Table 3, Table 4 are given in metres for readability. Because the absolute origin is application-dependent, the point clouds are stored as per-point coordinates and users are free to choose a frame appropriate to their use case. The complete repository is approximately 19 MB in size, with individual files sizes depicted in Fig. 1.

**Table 3:** Position of the LiDAR sensors in the main room.

LiDAR	X-coordinate (m)	Y-coordinate (m)	Z-coordinate (m)
Avia1	1.38	7.34	0.95
Avia2	1.70	10.33	0.97
Avia3	4.85	10.93	1.05
Avia4	8.24	8.73	1.08
Avia5	7.73	0.14	1.57
Avia6	3.81	0.24	1.44

**Table 4:** Position of the LiDAR sensors in the hallway.

LiDAR	X-coordinate (m)	Y-coordinate (m)	Z-coordinate (m)
Avia1	0.00	1.00	1.30
Avia2	3.70	16.95	1.30
Avia3	32.20	1.00	1.30
VLP1	3.70	0.50	1.30
VLP2	2.00	8.75	1.30

## 4 Experimental Design, Materials and Methods

### 4.1 Methods

This section describes the procedures and equipment used for data acquisition. First, the 3D measurements performed using LiDAR devices are presented. This is followed by a description of the RSSI measurements conducted using a commercial Wi-Fi access point and a user device.

### 4.2 Measurement system for 3D point cloud

The 3D point clouds of the indoor environment were captured using commercially available LiDAR sensors, including VLP-16 units from Velodyne (now Ouster) [16] and Avia units from Livox [17]. Measurements were conducted in two separate areas: the main room and the hallway.

In the main room, only the Livox Avia sensor was used. Its extended detection range and relatively high point density make it well suited for capturing detailed indoor scenes and accurately reconstructing furniture and structural elements. In the hallway, both Avia and VLP-16 sensors were deployed. The Avia sensors captured long-range details along the corridor with high density, while the VLP-16 sensors, offering a 360-degree horizontal field of view, complemented this coverage by capturing lateral areas that were not fully covered by the Avia sensors. The combination of both sensor types enabled comprehensive point cloud coverage, particularly in transitional areas such as doorways and corners. Overall, this sensor configuration was selected to leverage the strengths of each device: range and density from the Avia sensors and wide angular coverage from the VLP-16 sensors.

**Fig. 4:**
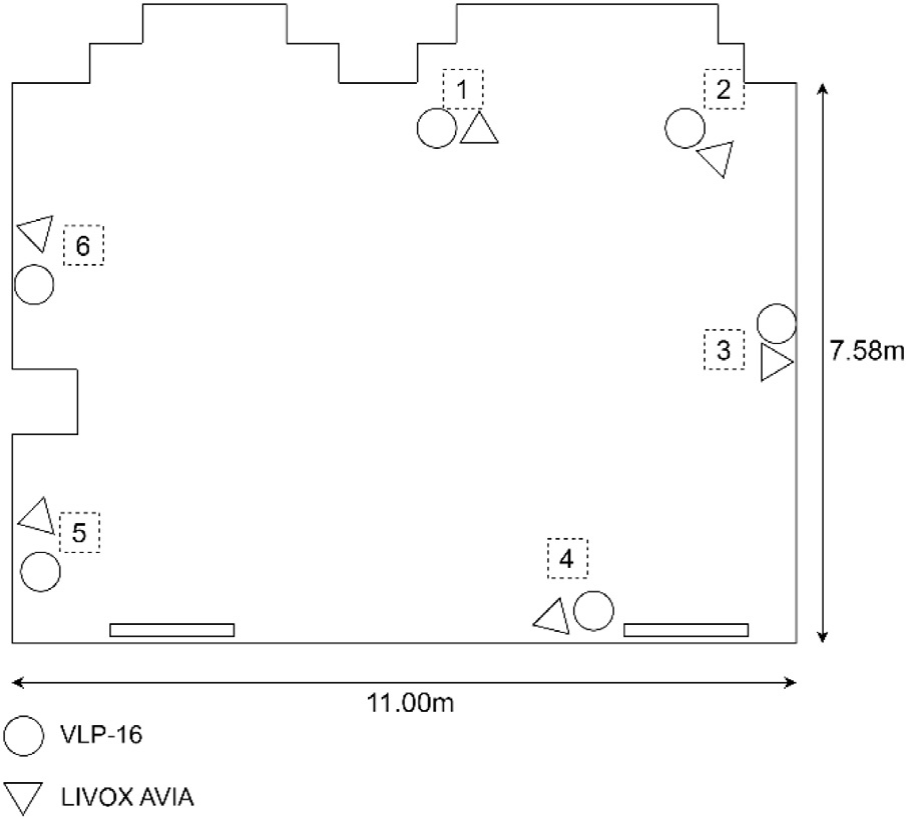
Room with position of LiDAR system.

Fig. 4 presents a plan-view layout of the open-concept main room, annotated with six LiDAR sensor stations positioned around the room’s perimeter. Each station is marked with a triangle indicating an Avia LiDAR sensor, while dashed squares indicate station numbering. The room is divided into two zones, a wood-surfaced area and a tiled area. Sensors were placed along all room boundaries to ensure complete coverage.

**Fig. 5:**
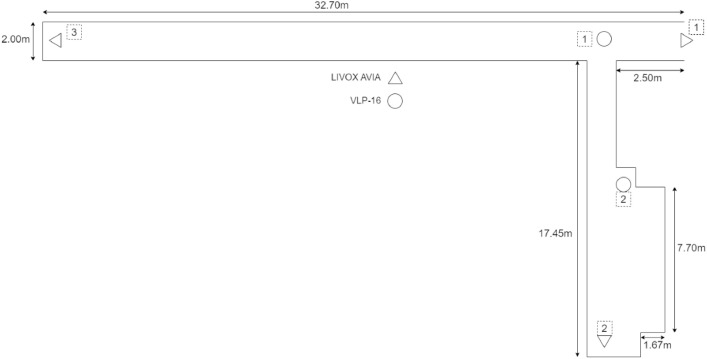
Hallway with position of LiDAR system.

Fig. 5 presents a plan-view layout of the hallway, annotated with three groups of LiDAR sensor stations. In this setup, Avia sensors are indicated by triangle markers and VLP-16 sensors by circular markers. The distribution of sensors ensures coverage across the entire corridor length.

Table 3 shows the sensor positions for the six Avia LiDAR sensors, installed in the stations 1 to 6 around the room in Fig. 4. Each row in the table corresponds to a single sensor, identified by its name in the “LiDAR” column. The X‐ and Y‐coordinates indicate the horizontal placement of the sensor in meters relative to an origin, which is in the bottom left corner, while the Z‐coordinate indicates the sensor’s height above the floor. These coordinates show that sensors are positioned at various points along the perimeter (X and Y-coordinate) and mounted at different heights (Z-coordinate) between roughly 0.9 m and 1.8 m, mostly on top of the furniture in the area. This distribution ensures coverage across the entire workspace and accommodates potential occlusions from furniture or architectural elements.

Table 4 shows the sensor positions for the five LiDAR sensors, three Avia units and two VLP units, installed in the stations 1 to 3 around the hallway in Fig. 5. Each row in the table corresponds to a single sensor, identified by its name in the “LiDAR” column. Similar to Table 2, the X‐ and Y‐coordinates indicate the horizontal placement of the sensor in meters relative to an origin, which is in the bottom left corner, while the Z‐coordinate indicates the sensor’s height above the floor. LiDAR sensors in the hallway were positioned on the special mounts and are all at the constant height of 1.30 m

In general, sensor mounting height influences the sensor’s field-of-view, incidence angles with surfaces, likelihood of occlusion by furniture or other obstacles, and ultimately the completeness of the point cloud collected. For instance, [18] identify mounting position as a factor for indoor LiDAR algorithm performance, but the influence was only noticed in occluded environments. We applied this reasoning to the furnished main-room environment and by selecting heights between 0.95 m and 1.57 m we reduced occlusion from furniture and improved coverage, whereas in the unobstructed corridor a uniform 1.30 m height was sufficient.

Per-station LiDAR scans were collected over an integration window of 90 s in the laboratory room and 150 s in the corridor. The acquisition pipeline ran on the Jetson Nano under JetPack 4.6, using our own implementation based on the methods described in [14] and [19]. Per-station scans within each room were merged following the same references, and the subsequent office-to-corridor registration was performed using the procedures detailed in [14] and [20]. The residual alignment error after registration is approximately ±3 cm for the VLP-16 sensors and ±2 cm for the Avia sensors. The LiDAR acquisition and multi-sensor registration infrastructure summarised here reuses the pipeline previously introduced in [14] and [20]. The novel contribution of the present work is the dedicated measurement campaign and the resulting paired 3D point-cloud and Wi-Fi RSSI dataset, which have not been released in any prior publication.

**Fig. 6:**
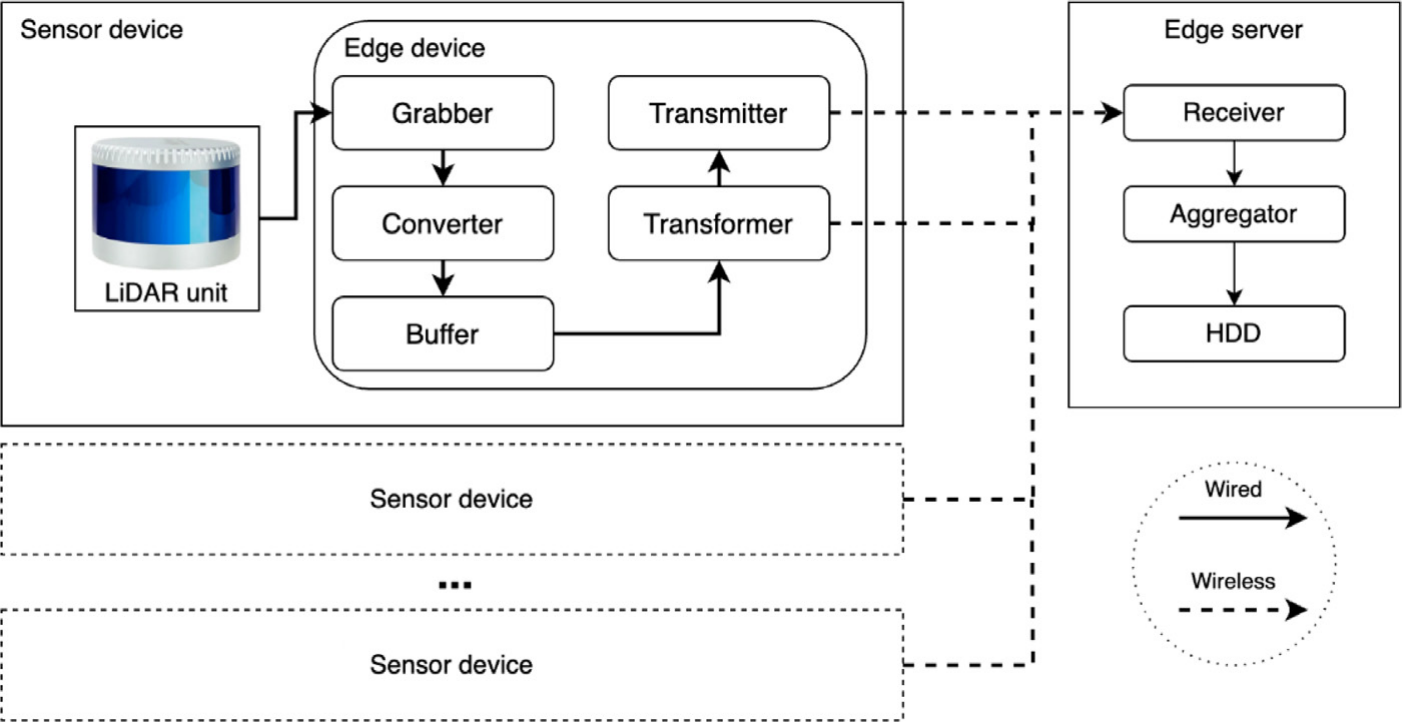
LiDAR sensor data acquisition scheme.

Fig. 6 presents the proposed LiDAR data acquisition and processing pipeline, highlighting both the hardware components and their interactions. In this system, each sensor device integrates a LiDAR unit (either Avia or VLP), which generates raw point clouds that are fed into the edge device in the form of NVIDIA Jetson Nano Developer Kit [21]. Within the edge device, a *grabber* module continuously retrieves frames from the LiDAR sensor, and a *converter* module reformats these frames into a standardized data representation. A *buffer* component is then employed to manage high-throughput bursts and mitigate potential network latency by providing temporary storage. Subsequently, a transformer module applies lightweight processing or compression steps to the data, optimizing it for transmission. The processed LiDAR data are then relayed via the transmitter module to the edge server through a TP-Link Archer AX73 router (configured as an AP) to facilitate reliable network connectivity. The edge server itself, implemented on an NVIDIA Jetson Xavier NX Developer Kit, receives incoming data through a *receiver* module, which hands the data off to an *aggregator* responsible for collating, synchronizing, or otherwise fusing data streams from multiple sensor devices. Finally, all integrated data are stored on a *hard disk drive* (HDD) in binary format to facilitate subsequent analysis.

### 4.3 Measurement system for RSSI

**Fig. 7:**
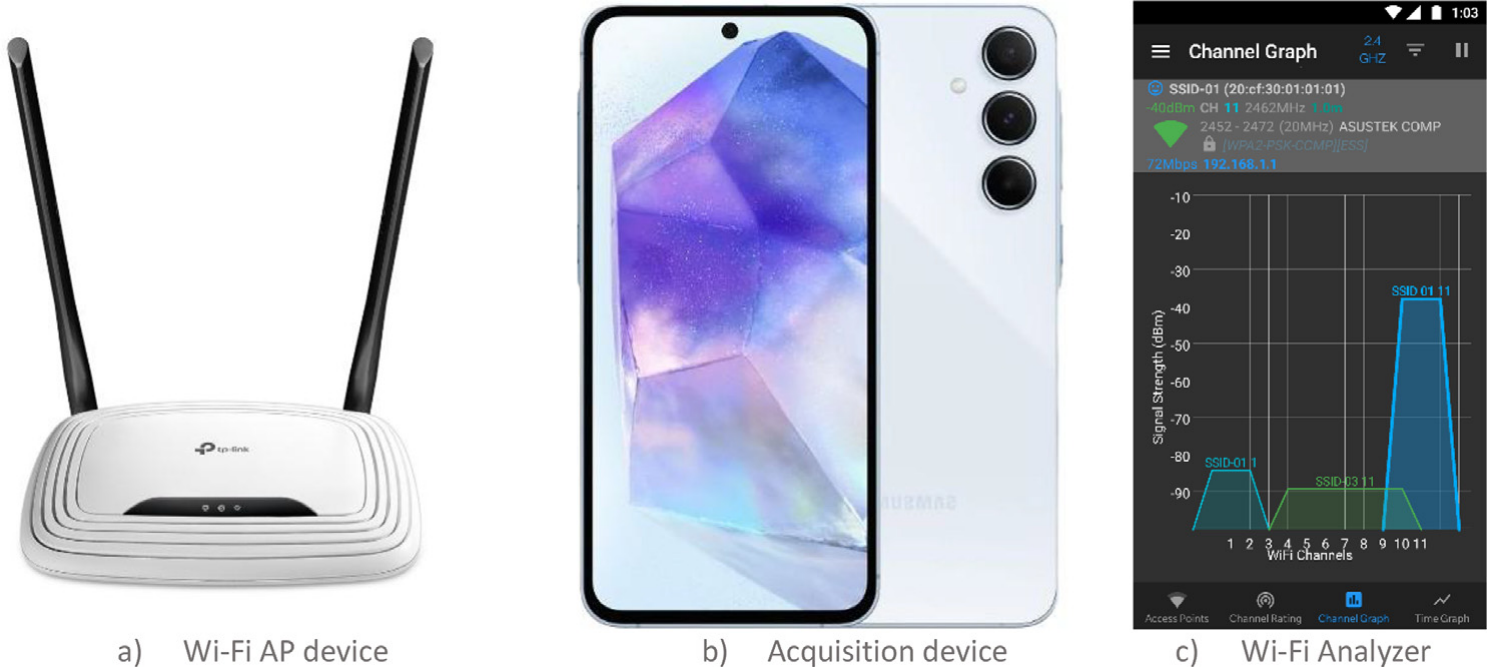
RSSI acquisition hardware and software.

Within the same indoor environment outlined in the previous section, RSSI data is collected at a set of predefined grid locations. The measurement setup employs a single TP-Link TL-WR841N device configured as a Wi-Fi AP, operating in the 2.4 GHz frequency band, as shown in Fig. 7. This AP is equipped with two omnidirectional antennas, which emit signals uniformly in all directions at the antenna’s elevation.

We collect the RSSI measurements using a commercial Android-based smartphone (Samsung model SM-A556B/DS, running Android 14) with the open-source application Wi-Fi Analyzer [22]. The use of widely available, off-the-shelf hardware for both the AP and the measurement device is a deliberate choice aimed at replicating realistic deployment conditions and capturing signal behavior under everyday usage scenarios. Other wireless networks, Wi-Fi and Bluetooth, are also operating in the same environment, however their influence is considered negligible [23], as well as distribution of values of Wi-Fi RSSI in the 2.4 GHz band of around 2 dBm [24], compared to the differences in spatial changes.

### 4.4 RSSI measurement process

The RSSI measurement process involves positioning a Wi-Fi AP at various locations across a predefined grid and collecting RSSI readings at each corresponding grid point. These readings are gathered using a User Equipment (UE) device in the form of a commercial smartphone held at an operating height of 1.5 m, reflecting typical user behavior. The AP is placed at a comparable height of 1.2 m to maintain consistency in signal propagation conditions.

For each measurement point and setup, a single reading was recorded from the Wi-Fi Analyzer application and stored without further processing. This means that the dataset captures spatial RSSI variation, but does not characterize short-term temporal variability at each measurement point. The AP operates on a single channel within the standard 2.4 GHz Wi-Fi band. The transmit power is fixed at 20 dBm by the manufacturer in compliance with applicable EU regulations and was not modified during the campaign. The AP’s two omnidirectional antennas radiate uniformly in the azimuthal plane, so antenna orientation is not a relevant degree of freedom in this setup. The mounting positions and heights of the AP and UE are described in the previous paragraph. Wi-Fi scan throttling was disabled on the measurement device (via Android developer options), ensuring that each reading reflects a fresh scan rather than a cached result. During each reading the smartphone was held with both hands in a natural orientation facing the operator, using a minimal-obstruction posture in which the operator avoided standing directly on the line between the AP and the UE.

The measurement locations are uniformly distributed throughout the indoor environment, as illustrated in Fig. 2a, which includes a point cloud representation of the space. An example grid of *53* measurement points is depicted in Fig. 3, where red dots indicate locations spaced *2 m* apart in both the *x* and *y* directions. The color variation of the contour plot illustrates the signal strength variation, *blue* marking regions with lower RSSI values, *yellow* marking regions with higher RSSI values. This spacing was chosen to balance the need for capturing meaningful variations in signal strength while minimizing redundancy and reducing the labor-intensive nature of manual measurements. Due to physical constraints of the office space, the RSSI measurement at location *6* (upper left corner), as an exception to the regular spacing, was taken only *1 m* from location *5*, as illustrated in Fig. 3.

The RSSI measurement points deviation is *0.2* *m*. Empirically it was observed that the RSSI values do not change for such small displacements, with the first observable changes noticed at around *1* *m* displacement. This is due to the limited sensitivity of devices, the software used, and the relatively low variability of the RSSI metric itself [25] for distances beyond *2* *m*. The RSSI values depend solely on the relative placement of the AP, the user equipment, and the intervening obstacles (office environment). Consequently, the dataset includes only the RSSI measurements and their associated spatial coordinates, while the temporal aspect is not considered relevant. Thus, in this setup, only spatial synchronization is ensured. The point number *1* is positioned at a distance of *0.2* *m* from both adjacent walls, in the room corner, and the points *2–6* along the wall at *0.2* *m* distance from it, therefore all other measurement points could be referenced to a common coordinate system derived from the 3D data.

The indoor environment comprises a typical office space (shown in orange in Fig. 2b) and an adjacent corridor and elevator hall extending southward (depicted in blue). This complex spatial layout serves two purposes: first, it simulates real-world Wi-Fi deployment scenarios; second, it enables the analysis of signal propagation under conditions involving multiple walls and significant spatial separation between the AP and measurement points. Measurement locations were chosen to represent areas of frequent user presence.

### 4.5 Measurement setup details

The measurement sequence is organized numerically, as illustrated in Fig. 3, to enable efficient navigation across the grid and minimize human error during data collection. A total of *20* distinct setups were created, each comprising *53* grid locations. While the expected number of RSSI samples is *1060* (*20 × 53*), the actual count is *1027* due to weak signals at distant points and occasional human error. These missing values can be estimated using preprocessing functions available in a public repository linked in this paper. In the visualizations, linear interpolation is used to fill the missing values using the *Scipy* [26] library. If needed for specific use case, other interpolation methods are also available for use, namely “nearest”, “slinear”, “cubic”, “quintic” and “pchip”.

The measurements were conducted under two general scenarios: one with the office completely empty of people, the furniture and room layout remaining unchanged, and another with regular working activity taking place with *7–10* people present. Each of the *20* setups (*12* in the first scenario and *8* in the second) was designed to reflect realistic signal propagation conditions while maximizing environmental diversity and experimental comparability. The aim is to provide data that would allow for analysis of how the presence of people during working days affects the signal coverage, compared to an unoccupied office.

#### 4.5.1 Scenario 1: empty office (12 setups)

In the first scenario, the office space was unoccupied, providing a baseline for signal measurements under static, interference-free conditions. Twelve different measurement setups were defined, each placing the AP at a different location in the room. The chosen positions include locations close to corners (e.g., positions 1 and 30), near the walls of the office (e.g., positions 3, 5, 18, 25), and along the corridor (e.g., positions 33, 37, 44). This arrangement allowed us to capture a wide range of propagation paths, including long-range line-of-sight (in the office and along the corridor), heavily obstructed links (points near the end of the corridor), and reflection-dominated geometries (along the corridor). Special attention was given to placing the AP in setups that reflect practical deployment norms, particularly along room perimeters, without introducing redundancy.

#### 4.5.2 Scenario 2: office with regular activity (8 setups)

With the second scenario, we aimed to capture the signal strength changes of indoor environments during regular office operations. Here, measurements were collected while *7* to *10* individuals were present in the room, engaged in normal activities such as working at desks, walking, or conversing. This scenario consists of two subgroups. In the first subgroup, four of the AP positions used in *Scenario 1* (specifically positions *1, 18, 26,* and *30*) were reused under occupied conditions. The presence of people affects the signal propagation, thus resulting in a new set of (lower dB) values besides the overlapping of the AP and UE locations in the same measurement positions as in *Scenario 1*. Therefore, the measurements obtained in these four setups are considered unique and new. Such data enables direct comparisons of signal behavior in identical spatial setups between empty and active environments, isolating the effects of human presence on signal attenuation.

In the second subgroup, four entirely new AP positions were introduced: *20, 14, 35,* and *47*. These were selected to represent functionally and structurally distinct zones within the office, such as densely populated desk clusters, regions near shared electronic equipment, and new locations along the corridor. These locations were not used in *Scenario 1* and serve to broaden the dataset by introducing previously unobserved signal dynamics. As such, *Scenario 2* provides grounds for the exploration and evaluation of potential REM models in new, unseen conditions.

Such a dataset composition ensures coverage of realistic deployment cases, supports comparative analyses, and enhances the representativeness of the measurement campaign for signal modeling and indoor localization tasks.

### 4.6 Technical validation

#### 4.6.1 Log-Distance path loss model

**Fig. 8:**
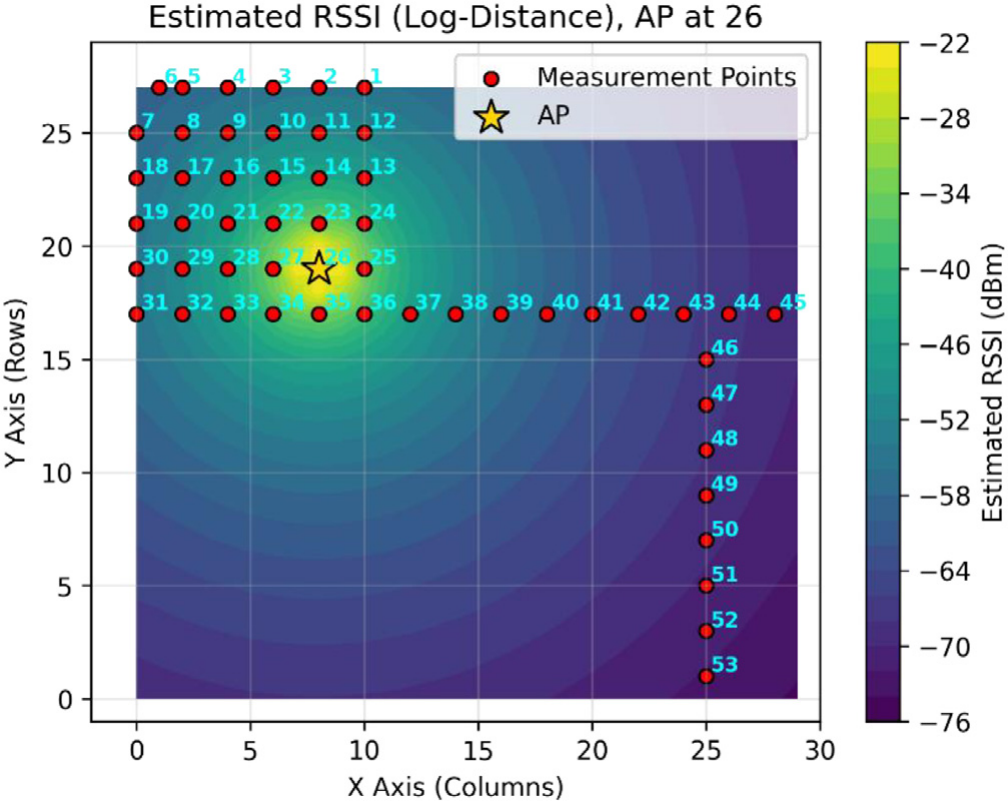
Logarithmic pathloss model estimation over the experimental indoor environment.

To assess the physical consistency of the collected RSSI measurements, we employ the log-distance path loss model as a reference estimator. This model is one of the most widely used empirical propagation models for indoor and outdoor wireless environments [27,28] and is particularly well-suited for indoor scenarios where a simple yet physically grounded characterization of signal decay with distance is required. The model expresses the path loss at distance *d* as:

$$\text{RSSI}\left(d\right)=\text{Pt}-\text{PL}\left(d_{0}\right)-10\cdot n\cdot \log_{10}\left(\frac{d}{d_{0}}\right)$$

with Pt set to the nominal transmit power of the Wi-Fi router (20 dBm), d_0_ = 1 m as the reference distance, and both the reference path loss PL(d_0_) and the path loss exponent *n* estimated per AP via ordinary least squares regression over the available measurement points. For each AP in the dataset, the model estimates the expected RSSI at any measurement location based solely on the Euclidean distance to the AP, using a fitted path loss exponent and a reference RSSI value at 1 m. Fig. 8 illustrates this for AP placed at position 26, where the estimated RSSI field is shown as a continuous heatmap over the measurement space. The concentric gradient, ranging from high RSSI (yellow, approximately 22 dBm) near the AP to low RSSI (dark purple, approximately 76 dBm) at far distances, reflects the expected monotonic decay with distance.

#### 4.6.2 Validation against literature-defined model

**Fig. 9:**
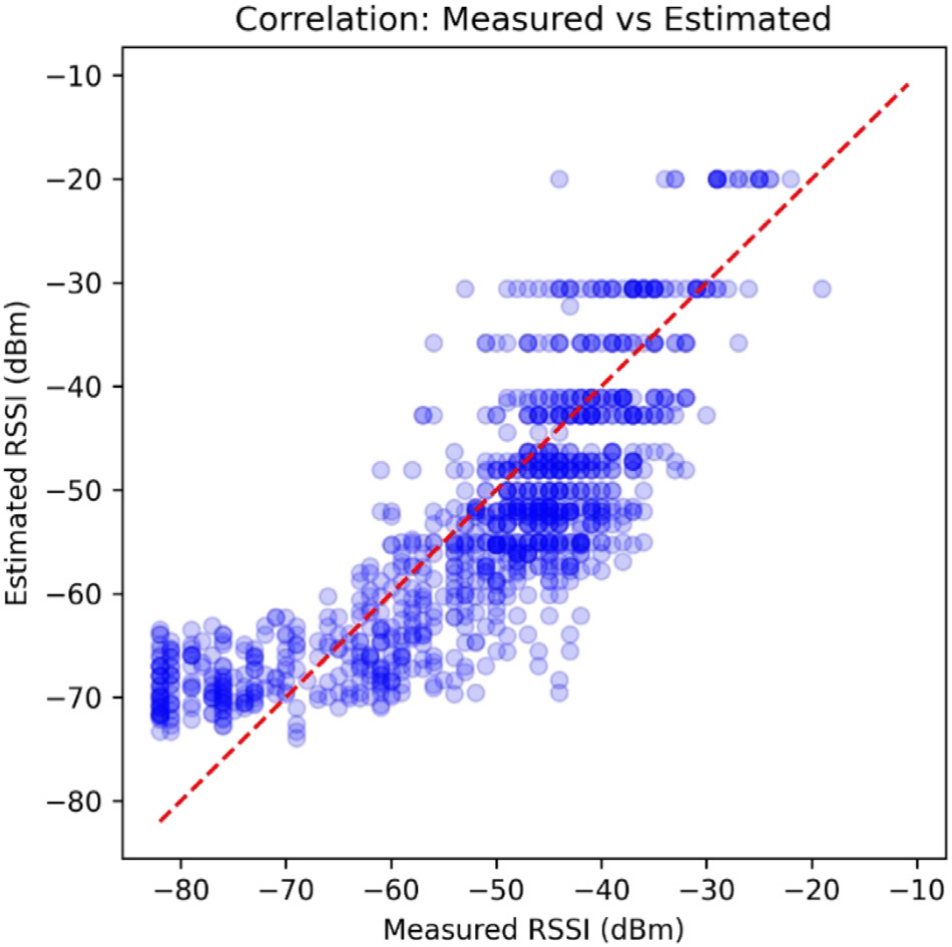
Correlation between literature-defined model and measured RSSI.

In the first validation stage, both *n* and PL(*d*_0_) are fixed to standard literature values rather than estimated from the acquired data, ensuring the validation is fully independent of the dataset itself. The path loss exponent is set to *n* = 3.5, consistent with values reported by [29,30] for multi-room indoor environments with partial wall obstruction, and the reference path loss is set to PL(*d*_0_) = 40 dB, corresponding to the free-space path loss at 1 m for the 2.4 GHz Wi-Fi band. The resulting correlation between measured and estimated RSSI values across all APs and measurement points confirms a clear positive linear trend, demonstrating that the measurements follow the expected distance-decay behaviour even when the model is not calibrated to the data. However, the plot reveals a non-monotonic bias structure across distance regimes. At far distances, corresponding to low measured RSSI values, the model tends to underestimate path loss, as the fixed exponent cannot account for the discrete attenuation introduced by walls between rooms. At mid-range distances, the model overestimates path loss, since *n* = 3.5 is conservative for same-floor, lightly obstructed paths. These systematic deviations, confirm that the measurements capture the full complexity of a multi-room indoor propagation environment in a physically interpretable manner. The observed bias pattern is consistent with the known limitations of a single-exponent log-distance model applied uniformly across environments with fundamentally different propagation regimes, and the deviations are coherent with the established indoor propagation findings [29] (Fig. 9).

#### 4.6.3 Dataset characterization via fitted path loss exponent

**Fig. 10:**
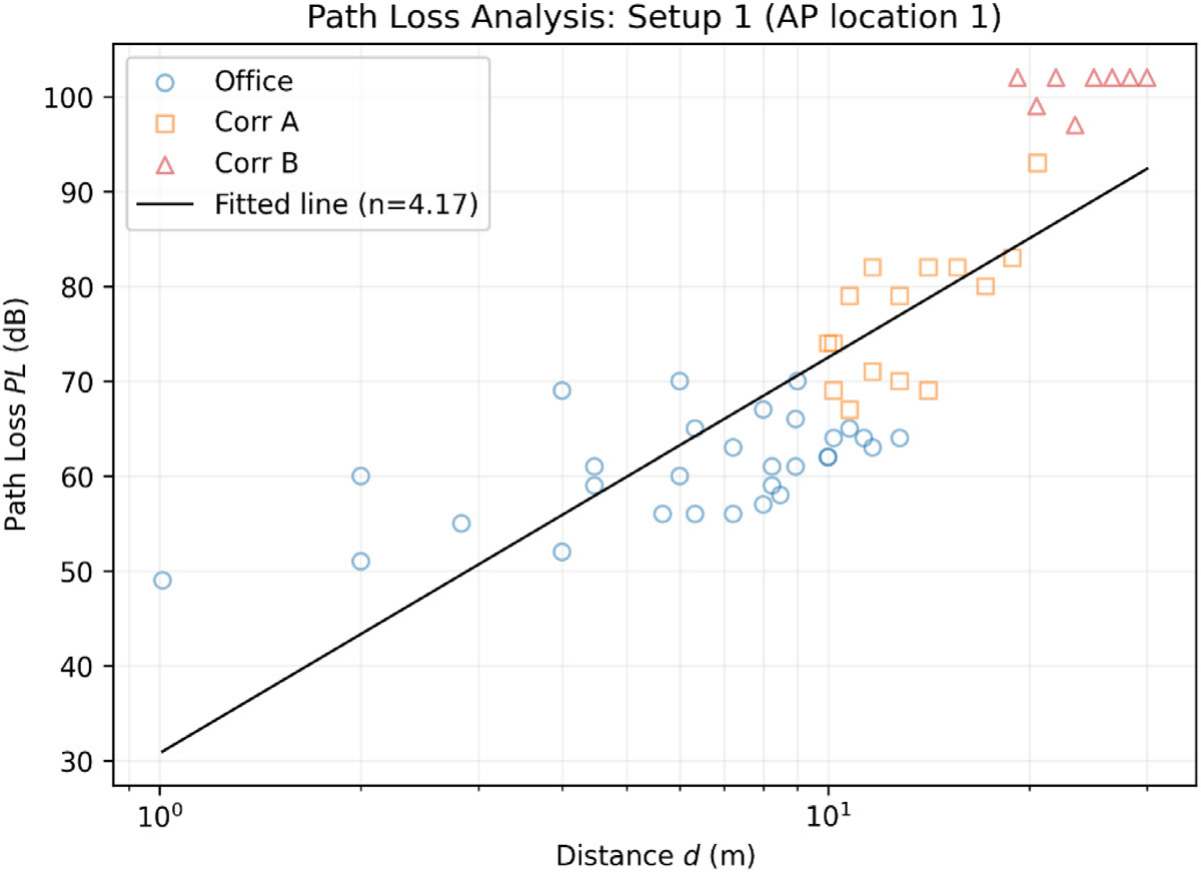
Example of fitted line for the exponent estimation based on Setup 1.

In the second stage, the model parameters PL(*d*_0_) and *n* are estimated per AP via ordinary least squares regression over all available measurement points for that AP [29]. Fitting is performed by rearranging the model as a linear system P_t_ − RSSI(*d*) = PL(*d*_0_) + 10 · *n* · log_10_(*d*/*d*_0_), where the left-hand side is computed from the measured RSSI values and the AP transmit power, and the right-hand side is expressed as a linear regression in log_10_(*d*/*d*_0_). The intercept and slope of this regression yield PL(*d*_0_) and *n* respectively. Example of the fitting result from the setup 1 is visualized on Fig. 10 where the different room points are show with different markers.

**Fig. 11:**
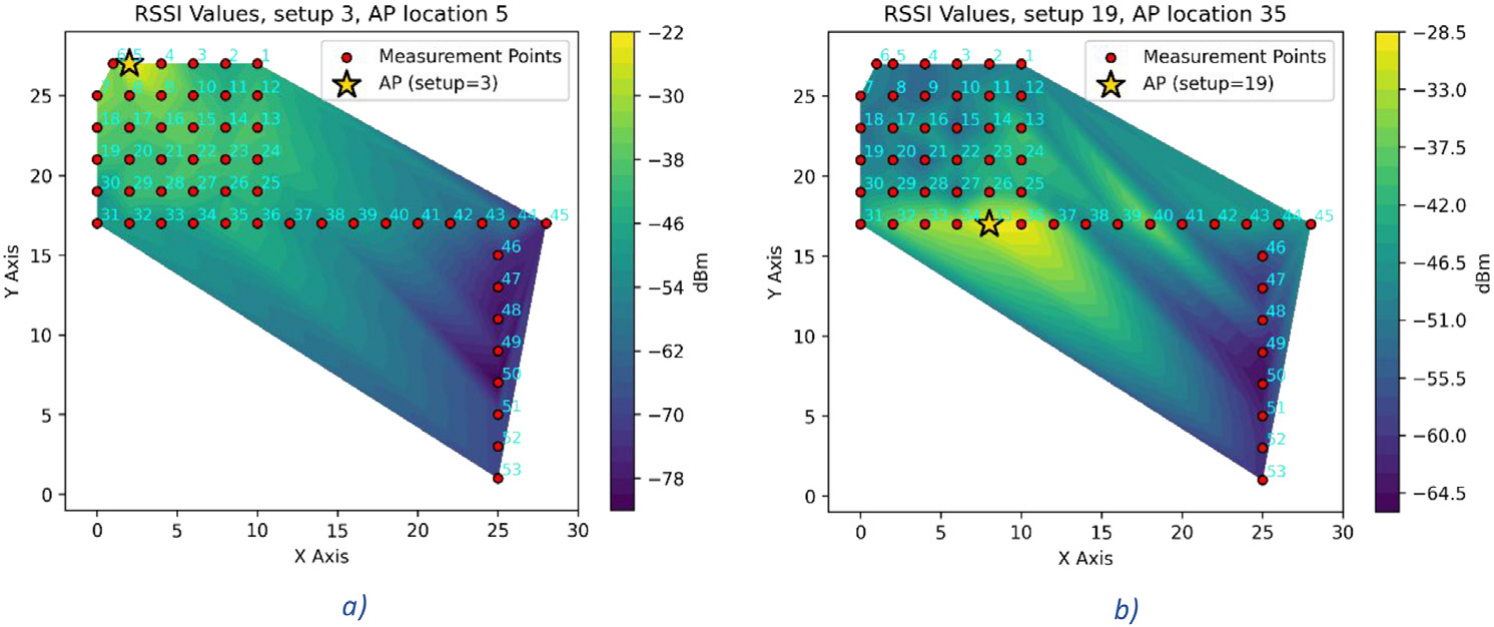
Example measurement setups.

The fitted path loss exponents across the 20 APs range from 1.89 to 4.17 and corresponds to the physical layout and measurement conditions of the environment. For APs 1 through 8, deployed in the office without occupants, the estimated values fall consistently in the range 2.83 to 4.17, with a group mean of approximately 3.49. This is in good agreement with the literature range for furnished indoor office environments [3] and reflects the combined attenuation contributions of office furniture, internal partitions, and the spatial distribution of measurement points across multiple rooms. Setups 13 through 18, recorded in the same office but with occupants present, yield a slightly higher group mean of approximately 3.57, consistent with the additional body shadowing introduced by human presence. For setups 9, 10, and 19, positioned in the corridor with the AP located along the corridor axis, the fitted exponents are markedly lower, at 1.92, 1.89, and 2.07 respectively. These near-free-space values are a direct consequence of the waveguiding effect, whereby signal propagation along an unobstructed corridor is channelled between the parallel walls, resulting in slower distance decay than in open or furnished spaces. This phenomenon is clearly visible in the corresponding heatmaps (e.g., Setup 19 at Fig. 11b), which show an elongated signal distribution along the corridor axis rather than the concentric decay pattern observed for office setups of AP (e.g. setup 3 in Fig. 11a). Finally, APs 19 and 20 in the corridor with occupants yield values of 2.07 and 3.76. The wide variation in the second corridor could be attributed to the multiple walls existing between the AP, which is in the second corridor, and the measurement points the office space. Taken together, the distribution of fitted *n* values across all 20 APs is physically coherent, spatially interpretable, and consistent with established indoor propagation literature.

A complete summary of the fit quality for each of the 20 setups is provided in the table below, listing the AP location, the room and occupancy state, the fitted path-loss exponent (*n*), the reference path loss at *d_0_ (PL(d_0_), (dB)*, the residual RMSE (*dB*), the coefficient of determination (*R^2^*), and the number of measured points used in the fit. The same table is deposited in the Zenodo record as setups_metadata.csv to support independent verification of the fitted path-loss parameters and validation results (Table 5).

**Table 5:** Per-setup fitted log-distance path-loss parameters and fit quality. n = path-loss exponent; PL(d0) = reference path loss at d0 = 1 m; RMSE and R² computed on measured-vs-fitted path loss across the 53 measurement points per setup.

Setup	AP loc.	Room	Occupancy	n	PL(d0) (dB)	RMSE (dB)	R²	N pts
1	1	Office	empty	4.17	30.77	8.93	0.659	53
2	3	Office	empty	3.57	35.95	6.96	0.758	53
3	5	Office	empty	3.23	37.13	6.68	0.764	53
4	18	Office	empty	3.06	39.54	6.20	0.764	53
5	30	Office	empty	2.83	42.79	6.87	0.676	53
6	26	Office	empty	3.57	42.48	8.82	0.626	53
7	25	Office	empty	3.51	36.18	9.61	0.538	53
8	12	Office	empty	4.01	33.78	7.65	0.725	53
9	33	Corridor A	empty	1.92	48.96	4.82	0.628	53
10	37	Corridor A	empty	1.89	52.61	5.27	0.466	53
11	44	Corridor A	empty	2.11	50.59	5.92	0.583	53
12	49	Corridor B	empty	4.17	35.62	6.13	0.837	53
13	1	Office	occupied	3.86	34.44	7.70	0.690	53
14	18	Office	occupied	3.24	39.83	6.36	0.776	53
15	26	Office	occupied	3.55	42.73	9.09	0.610	53
16	30	Office	occupied	3.26	40.74	8.48	0.644	53
17	20	Office	occupied	3.45	39.93	6.58	0.791	53
18	14	Office	occupied	4.09	37.40	5.92	0.843	53
19	35	Corridor A	occupied	2.07	49.26	5.21	0.585	53
20	47	Corridor B	occupied	3.76	39.51	5.71	0.830	53

Beyond the quantitative path-loss analysis, several qualitative observations help interpret the measured RSSI fields and serve as useful sanity checks for models trained on this dataset. First, comparing Setup 1 (AP at position 1, empty office) with Setup 13 (AP at position 1, occupied office) isolates the effect of human presence on a fixed AP location. The signal strength at interior office points is noticeably lower in the occupied scenario, consistent with absorption and scattering by human bodies. Furthermore, in several setups a relatively weaker or comparable RSSI is observed at the AP’s exact location compared with immediately adjacent positions, for example, in Setup 2 the AP is placed at position 3 where the recorded RSSI is approximately −33 dBm, whereas the neighbouring position 2 shows a significantly stronger signal of approximately −19 dBm. This pattern is consistent with the vertical proximity of the UE to the AP placing it within the antenna’s radiation null: omnidirectional dipole antennas typically exhibit a “donut-shaped” radiation pattern that produces reduced gain directly above or below the element. A similar pattern is visible in Setup 19 (AP at position 35), where the adjacent position 36 records a comparable RSSI of −29 dBm. In the narrow corridor geometry this effect is further shaped by multipath and surface reflections that produce localised signal enhancements. Together, these observations demonstrate that the dataset captures not only the average distance-decay behaviour targeted by the fitted path-loss model but also higher-order phenomena (body shadowing, antenna-pattern nulls, corridor waveguiding and multipath) that are relevant for training and evaluating realistic REM estimators.

Given the dataset’s spatial consistency between the RSSI measurements and the 3D representation of the environment, it is well suited to training and validating REM estimation models. Unlike earlier works that rely on 2D input images annotated with AP locations, this dataset provides 3D input data that captures the detailed environmental structure along with actual RSSI measurements serving as ground truth for REM estimation. While purely simulated data can support large-scale initial training, the present dataset enables realistic validation in a multi-room indoor setting, and its coverage of both human-present and human-free conditions supports systematic evaluation of model robustness to occupancy variability. The dataset is organised to support progressive validation, starting with pre-deployment testing on the empty-office setups, continuing with deployment fine-tuning on the occupied-office setups that reuse Scenario 1 positions, and concluding with evaluation on the four Scenario 2 setups that introduce previously unseen AP positions.

## 5 Usage Notes

The intended use of the dataset is to support the development of advanced machine learning models, including those based on generative AI, for accurate REM estimation from 3D environmental data, correlating spatial signal measurements with the physical structure of the environment. This is particularly relevant for applications that demand consistently high throughput and ultra-low latency, such as XR-related devices. The dataset additionally enables analysis of how human presence during working days affects indoor Wi-Fi coverage. Although the number of people present and their uncontrolled activities (walking, sitting, conversing) influence signal propagation, these factors were not controlled in the current study, and will be considered in future work.

The dataset files, including the RSSI measurements, 3D point clouds, and accompanying software, are contained in a single archive retrievable from the Zenodo record. After downloading, users can open the project in an IDE of their choice (for example PyCharm) and install the required libraries listed in requirements.txt. Once installed, the example_operations.ipynb notebook provides sample workflows for loading and processing both data types end-to-end.

The point cloud data is provided in two separate files, one for the office and one for the corridor, in addition to the combined registered file. Because the RSSI measurements are environment-specific, the dataset supports multiple usage configurations. In simpler scenarios, researchers can use only the office data to study signal propagation in open indoor areas with and without human presence. Alternatively, the corridor data can be used independently to analyse long line-of-sight behaviour and reflection-rich conditions in a narrow (approximately 2 m wide) waveguide-like geometry. More complex experiments can use the full combined dataset, including cases with multiple walls between the AP and UE, to study challenging multipath and obstruction scenarios.

To support manual inspection and alignment of the 3D point clouds, the repository provides a lightweight interactive toolbox built on the Open3D library. The toolbox allows users to visually crop regions of interest within point clouds, select key correspondence points across multiple scans, perform rigid registration based on those manual correspondences, inspect the registration result in real time, and export the aligned point cloud for further use.

For the Wi-Fi side, the toolbox provides a set of functions for loading, organising, and visualising the RSSI measurements collected across the 20 experimental setups. Signal strength values are parsed into a unified tabular format and spatially mapped onto the predefined two-dimensional grid that reflects the physical layout of the environment (single office room plus two corridors). The toolbox handles missing or corrupted entries through interpolation (six selectable methods) to maintain data consistency when required for downstream processing. For visualisation, it generates contour plots illustrating the spatial signal distribution and measurement density, along with annotations marking the measurement indices and AP locations. Contour plots are generated using the matplotlib library via the tricontourf function, based on linear interpolation, and are intended for visualisation purposes only; alternative interpolation methods can be used for quantitative work. For reproducibility and efficient storage, the processed datasets, including signal maps, setup identifiers, and spatial indices, are compiled into compressed HDF5 files with timestamped filenames.

## Limitations

The dataset described in this paper provides a useful and well-controlled benchmark for studying RSSI behaviour in an indoor office environment, however, our work also indicates several directions for future extension. First, the measurement environment is confined to a single indoor setting comprising one office and two adjacent corridors, which provides a focused basis for analysis, although additional measurements in other building types, floor plans, or construction materials would further improve the generalizability of the data. Second, the dataset captures two distinct occupancy conditions, namely an office with and without people, but does not systematically vary occupancy levels, meaning that the effect of different crowd densities on signal propagation remains an important direction for future study. Third, RSSI measurements are inherently subject to hardware-specific variability, as the received signal strength depends not only on propagation conditions but also on antenna gain patterns, receiver sensitivity, and firmware-level averaging, none of which are fully controllable across different collection sessions, although such effects are also representative of practical deployment conditions. Fourth, each measurement point is represented by a single recorded reading per setup. Therefore, the dataset does not characterise temporal variability between points, short-term fading, or measurement uncertainty from repeated samples.

## Ethics Statement

The authors confirm that they have read and follow the ethical requirements for publication in *Data in Brief* and that the current work does not involve human subjects, animal experiments, or any data collected from social media platforms. Although some RSSI measurements were collected during regular office activity with people present in the environment, no personal, biometric, visual, audio, behavioural, or identifying information was collected. Human presence was considered only as an environmental condition affecting wireless signal propagation.

## CRediT Author Statement

**Ljupcho Milosheski:** Conceptualization, Methodology, Software, Validation, Formal Analysis, Investigation, Data Curation, Writing - Original Draft. **Kuon Akiyama:** Methodology, Software, Data Curation. **Blaž Bertalanič:** Validation, Investigation, Writing - Original Draft. **Jernej Hribar:** Resources, Writing – Review & Editing, Supervision, Funding Acquisition. **Ryoichi Shinkuma:** Resources, Writing – Review & Editing, Supervision, Funding Acquisition.

## Data Availability

ZenodoAn Indoor Radio Mapping Dataset Combining 3D Point Clouds and RSSI (Original data).

## Footnotes

1 https://doi.org/10.5281/zenodo.20411480.

## References

[1] Ian F.A., Hongzhi G. (2022). Wireless communication research challenges for extended reality (XR). ITU J. Future Evolv. Technol..

[2] Rhode S. (2023). The metaverse and extended reality – implications for wireless communications. Rohde-Schwar..

[3] Pesko M., Javornik T., Kosir A., Stular M., Mohorcic M. (2014). Radio environment maps: the survey of construction methods. KSII Trans. Int. Inform. Syst. (TIIS).

[4] YR. Inc., “Wireless InSite propagation software,” [Online]. Available: https://www.remcom.com/wireless-insite-propagation-software. [Accessed 17 June 2025].

[5] Hoydis J., Cammerer S., Ait A.F., Nimier-David M., Maggi L., Marcus G., Vem A., Keller A. (2022). Sionna.

[6] Rufaida S., Leu J.-S., Su K.-W., Takada J.-I. (2020). Construction of an indoor radio environment map using gradient boosting decision tree. Wireless Netwo..

[7] Wang X., Tao K., Cheng N., Yin Z., Li Z., Zhang Y. (2025). Radiodiff: an effective generative diffusion model for sampling-free dynamic radio map construction. IEEe Trans. Cogn. Commun. Netw..

[8] Quan H., Ni W., Zhang T., Ye X., Xie Z., Wang S. (2025). Large language model agents for radio map generation and wireless network planning. IEEE Network. Lett..

[9] Li X., Li H., Li X., Zhu G., Qi N., Xiao M. (2025). 2025 IEEE Wireless Communications and Networking Conference (WCNC).

[10] Chen Q., Yang J., Huang M., Zhou Q. (2024). Act-gan: radio map construction based on generative adversarial networks with act blocks. IET Commun..

[11] Jaensch F., Caire G., Demir B. (2024). http://arXiv:2402.00878.

[12] Bakirtzis S., Chen J., Qiu K., Zhang J., Wassell I. (2022). Em deepray: an expedient, generalizable, and realistic data-driven indoor propagation model. IEEe Trans. Antennas. Propag..

[13] Cisse C.T., Guillet V., Baala O., Spies F., Caminada A. (2024). 2024 18th European Conference on Antennas and Propagation (EuCAP).

[14] Akiyama K., Azuma K., Shinkuma R., Shiomi J. (2023). Real-time adaptive data transmission against various traffic load in multi-LIDAR sensor network for indoor monitoring. IEEE Sens. J..

[15] Nimier-David M., Vicini D., Zeltner T., Jakob W. (2019). Mitsuba 2: a retargetable forward and inverse renderer. ACM Trans. Graph. (ToG).

[16] Ouster, “ouster.com,” Ouster, [Online]. Available: https://ouster.com/products/hardware/vlp-16. [Accessed 12 February 2026].

[17] Livox, “livoxtech.com,” LivoxTech, [Online]. Available: https://www.livoxtech.com/avia/specs. [Accessed 12 February 2026].

[18] Garigipati B., Strokina N., Ghabcheloo R. (2022). 2022 25th International Conference on Information Fusion (FUSION).

[19] Li C., Akiyama K., Shinkuma R., Sato S., Nobukiyo T., Iwai T., Hamada K. (2021). IEEE International Conference on Communications Workshops (ICC Workshops).

[20] Azuma K., Akiyama K., Shinkuma R., Trovato G., Nihei K., Iwai T. (2023). Estimation of spatial features in 3-D-sensor network using multiple LiDARs for indoor monitoring. IEEE Sens..

[21] NVIDIA (2025). Jetson Nano developer Kit. NVIDIA.

[22] VREM Software Development (2025). WiFiAnalyzer. https://github.com/VREMSoftwareDevelopment/WiFiAnalyzer.

[23] Garroppo R., Gazzarrini L., Giordano S., Tavanti L. (2011). 2011 IEEE International Symposium on a World of Wireless, Mobile and Multimedia Networks.

[24] Park J.-G., Curtis D., Teller S., Ledlie J. (2011). 2011 Proceedings IEEE INFOCOM.

[25] Sadowski S., Spachos P. (2018). Rssi-based indoor localization with the internet of things. IEEe Access..

[26] P.e.a. Virtanen, “SciPy 1.0: fundamental algorithms for scientific computing in Python,” ArXiv., 2019. 10.1038/s41592-019-0686-2 PMC7056644 32015543

[27] G. A. (2005).

[28] Rappaport T. (2002).

[29] Scott Y.S., Rappaport T.S. (1992). 914 MHz path loss prediction models for indoor wireless communications in multifloored buildings. IEEe Trans. Antennas. Propag..

[30] Robert A., Dinesh T., Xinrong L. (2006). Wireless Networks and Emerging Technologies.

